# Redetermination of aqua­(dihydrogen ethyl­enediamine­tetra­acetato-κ^5^ *O*,*O*′,*N*,*N*′,*O*′′)nickel(II)

**DOI:** 10.1107/S1600536810002011

**Published:** 2010-01-23

**Authors:** Ivana Kočanová, Juraj Kuchár, Vladimíra Dankovičová, Juraj Černák

**Affiliations:** a Department of Inorganic Chemistry, Institute of Chemistry, P. J.Šafárik University, Moyzesova 11, 041 54 Košice, Slovakia

## Abstract

The crystal structure of the title compound, [Ni(C_10_H_14_N_2_O_8_)(H_2_O)] or [Ni(H_2_edta)(H_2_O)] (H_4_edta is ethyl­ene­diamine­tetra­acetic acid), originally determined by Smith & Hoard [*J. Am. Chem. Soc.* (1959), **81**, 556–561] has been redetermined to a significantly higher precision. The Ni^II^ atom is coordinated in a distorted octa­hedral geometry by two N atoms and three O atoms from three carboxyl­ate groups of the H_2_edta^2−^ ligand and by an O atom of a water mol­ecule. The complex mol­ecules are linked by inter­molecular O—H⋯O hydrogen bonds into layers perpendicular to [100].

## Related literature

For the crystal structures of nickel(II) complexes with deprotonized derivates of H_4_edta, see: Agre, Trunov *et al.* (1980 ▶); Agre, Sysoeva *et al.* (1980 ▶); Agre *et al.* (1981 ▶); Coronado *et al.* (1986 ▶); Sysoeva *et al.* (1981 ▶); Porai-Koshits *et al.* (1975 ▶); Sysoeva *et al.* (1986 ▶); Zubkowski *et al.* (1995 ▶); Stephens (1969 ▶). For the earlier determination of the title compound, see: Smith & Hoard (1959 ▶).
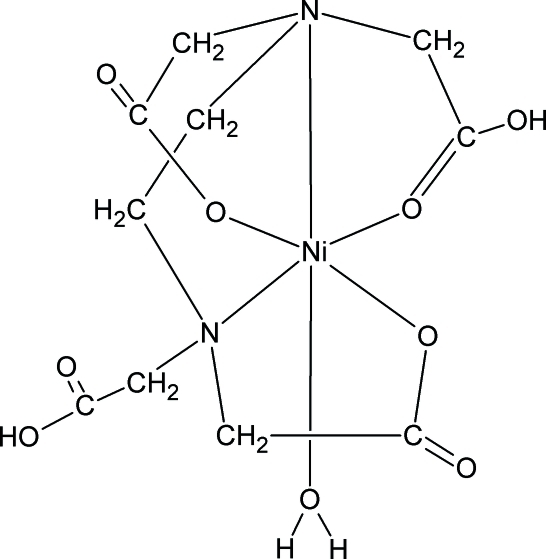


## Experimental

### 

#### Crystal data

[Ni(C_10_H_14_N_2_O_8_)(H_2_O)]
*M* *_r_* = 366.96
Monoclinic, 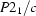

*a* = 11.6786 (2) Å
*b* = 6.9358 (1) Å
*c* = 16.6343 (2) Å
β = 91.140 (1)°
*V* = 1347.12 (3) Å^3^
*Z* = 4
Mo *K*α radiation
μ = 1.49 mm^−1^
*T* = 291 K
0.35 × 0.31 × 0.15 mm

#### Data collection

Oxford Diffraction Xcalibur diffractometer with a Sapphire2 detector
Absorption correction: numerical [Clark & Reid (1995 ▶) in *CrysAlis PRO* (Oxford Diffraction, 2009 ▶)] *T*_min_ = 0.690, *T*_max_ = 0.830
43864 measured reflections
2935 independent reflections
2534 reflections with *I* > 2σ(*I*)
*R*_int_ = 0.030

#### Refinement

*R*[*F* ^2^ > 2σ(*F* ^2^)] = 0.020
*wR*(*F* ^2^) = 0.055
*S* = 1.06
2935 reflections
198 parameters
H-atom parameters constrained
Δρ_max_ = 0.33 e Å^−3^
Δρ_min_ = −0.18 e Å^−3^

### 

Data collection: *CrysAlis PRO* (Oxford Diffraction, 2009 ▶); cell refinement: *CrysAlis PRO*; data reduction: *CrysAlis PRO*; program(s) used to solve structure: *SHELXS97* (Sheldrick, 2008 ▶); program(s) used to refine structure: *SHELXL97* (Sheldrick, 2008 ▶); molecular graphics: *DIAMOND* (Crystal Impact, 2007 ▶); software used to prepare material for publication: *SHELXL97*.

## Supplementary Material

Crystal structure: contains datablocks I, global, New_Global_Publ_Block. DOI: 10.1107/S1600536810002011/rz2407sup1.cif

Structure factors: contains datablocks I. DOI: 10.1107/S1600536810002011/rz2407Isup2.hkl

Additional supplementary materials:  crystallographic information; 3D view; checkCIF report

## Figures and Tables

**Table 1:** Hydrogen-bond geometry (Å, °)

*D*—H⋯*A*	*D*—H	H⋯*A*	*D*⋯*A*	*D*—H⋯*A*
O1—H1⋯O7^i^	0.82	1.77	2.5557 (14)	159
O3—H3⋯O5^ii^	0.82	1.79	2.5864 (13)	164
O6—H6*A*⋯O9^iii^	0.88	2.15	2.9294 (12)	148
O6—H6*B*⋯O2^iv^	0.86	1.81	2.6394 (11)	162

Symmetry codes: (i) 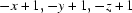; (ii) 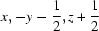; (iii) 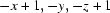; (iv) 
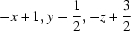
.

**Fig. 1.**
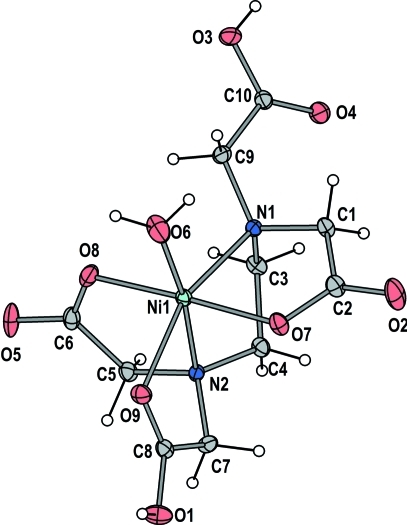
The molecular structure of the title molecule with the atom-numbering scheme. Displacement ellipsoids are drawn at the 30% probability level.

**Fig. 2.**
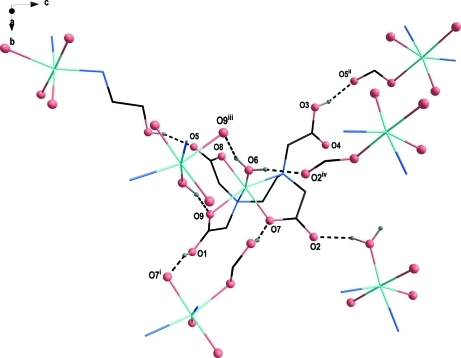
View of the hydrogen bonding network in the title compound. Hydrogen atoms not involved in hydrogen bonding are omitted for clarity. Symmetry codes: (i) 1 - x,1 - y,1 - z; (ii) x,-0.5 - y,1/2 + z; (iii) 1 - x,-y,1 - z; (iv) 1 - x,-1/2 + y,1.5 - z

**Fig. 3.**
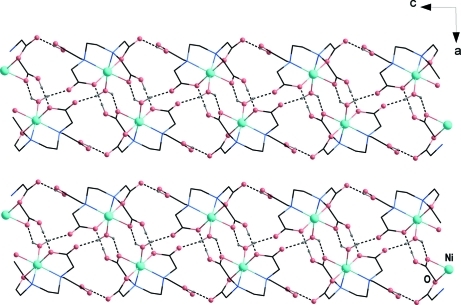
Hydrogen bonded layers in the crystal structure of the title compound. Hydrogen atoms not involved in hydrogen bonding are omitted for clarity.

## supplementary crystallographic information

### Comment

We are interested in the synthesis and characterization of Cu—Ni heterobimetallic complexes as models of magnetic alternate chains. Few Cu—Ni heterobimetallic complexes based on edta-type ligands (H_4_edta = ethylenediaminetetraacetic acid) have been already structurally characterized (Agre, Trunov *et al.*, 1980; Agre, Sysoeva *et al.*, 1980; Agre *et al.*, 1981). Previously, the crystal structures of one-dimensional [Ni(H_2_O)_4_(edta)Ni].2H_2_O (Coronado *et al.*, 1986), dinuclear [Ni_2_(en)(edta)(H_2_O)_3_].H_2_O (Sysoeva *et al.*, 1981), ionic [Ni(H_2_O)_6_][Ni(Hedta)]_2_.2H_2_O (Porai-Koshits *et al.*,  1975) and ionic [Ni(en)_3_][Ni(edta)].4H_2_O (Sysoeva *et al.*, 1986) complexes with the edta(4-) ligand were reported. As a part of our synthetic experiments on Cu—Ni heterobimetallic complexes from the aqueous system Ni^2+^-H_4_edta we have isolated the title compound. Its crystal structure was already studied by Smith & Hoard (1959). They obtained a correct structural model, however only two common isotropic thermal parameters were used and the positions of the hydrogen atoms were not determined. Consequently, the precision of the geometric parameters was limited.

In the crystal structure of the title compound (Fig. 1) the nickel(II) atom exhibits an elongated octahedral coordination geometry. Five coordinations sites are occupied by the partially deprotonized, pentadentate chelate H_2_edta^2-^ ligand, which coordinates through both nitrogen atoms and three oxygen atoms from carboxylate groups, among these two are deprotonized. The sixth coordination site is occupied by the oxygen atom of a water molecule. The fourth carboxylate group is not coordinated to the metal. The same type of coordination provided by the H_2_edta^2-^ ligand was observed *e.g.* in analogous complexes [Co(H_2_edta)(H_2_O)].H_2_O (Zubkowski *et al.*, 1995) and [Cu(H_2_edta)(H_2_O)] (Stephens, 1969).  As expected, the intrachelate angles D1—Ni—D2 (D's are N and O donor atoms) in the title compound are significantly smaller then the ideal value of 90°. In the crystal packing, the H atoms of the water molecule and of two carboxylic groups are involved in intermolecular O—H···O hydrogen bonds (Fig. 2; Table 1) forming layers perpendicular to [100] (Fig. 3).

### Experimental

Chemicals of reagent grade quality were obtained from commercial sources and were used as received. Solid NiCO_3_.2Ni(OH)_2_ (0.304 g, 1 mmol), ethylenediaminetetraacetic acid (0.877 g, 3 mmol) and tetraethylammonium bromide (0.421 g, 2 mmol) were dissolved under stirring  in 10 cm^3^ of water at room temperature. The formed blue solution was filtered and left aside for crystallization at room temperature. After eight months, few blue prismatic crystals of the title compound appeared along with white microcrystalline material on slow evaporation of the solvent.

### Refinement

In order to allow a direct comparison of the present crystal structure determination with the previously published one (Smith & Hoard, 1959) the same labelling of the atoms was adopted. The hydrogen atoms of the water molecule were located in difference Fourier map, and refined with the O—H bond and the H···H separation restrained to 0.85 and 1.380 Å, respectively, and with *U*_iso_(H) = 1.5 *U*_eq_(O). The hydrogen atoms of the H_2_edta^2-^ ligand were positioned geometrically with C—H = 0.97 Å and constrained to ride on their parent atoms with *U*_iso_(H) = 1.2*U*_iso_(C).

### Crystal data

**Table:**

[Ni(C_10_H_14_N_2_O_8_)(H_2_O)]	*F*(000) = 760
*M**_r_* = 366.96	*D*_x_ = 1.809 Mg m^−^^3^
Monoclinic, *P*2_1_/*c*	Mo *K*α radiation, λ = 0.71073 Å
Hall symbol: -P 2ybc	Cell parameters from 23028 reflections
*a* = 11.6786 (2) Å	θ = 2.9–29.7°
*b* = 6.9358 (1) Å	µ = 1.49 mm^−^^1^
*c* = 16.6343 (2) Å	*T* = 291 K
β = 91.140 (1)°	Prism, blue
*V* = 1347.12 (3) Å^3^	0.35 × 0.31 × 0.15 mm
*Z* = 4	

### Data collection

**Table:**

Oxford Diffraction Xcalibur diffractometer with a Sapphire2 (large Be window) detector	2935 independent reflections
Radiation source: fine-focus sealed tube	2534 reflections with *I* > 2σ(*I*)
graphite	*R*_int_ = 0.030
Detector resolution: 8.3438 pixels mm^-1^	θ_max_ = 27.0°, θ_min_ = 3.0°
ω scans	*h* = −14→14
Absorption correction: numerical [Clark & Reid (1995) in *CrysAlis PRO* (Oxford Diffraction, 2009)]	*k* = −8→8
*T*_min_ = 0.690, *T*_max_ = 0.830	*l* = −21→21
43864 measured reflections	

### Refinement

**Table:**

Refinement on *F*^2^	Primary atom site location: structure-invariant direct methods
Least-squares matrix: full	Secondary atom site location: difference Fourier map
*R*[*F*^2^ > 2σ(*F*^2^)] = 0.020	Hydrogen site location: inferred from neighbouring sites
*wR*(*F*^2^) = 0.055	H-atom parameters constrained
*S* = 1.06	*w* = 1/[σ^2^(*F*_o_^2^) + (0.0343*P*)^2^ + 0.1019*P*] where *P* = (*F*_o_^2^ + 2*F*_c_^2^)/3
2935 reflections	(Δ/σ)_max_ < 0.001
198 parameters	Δρ_max_ = 0.33 e Å^−^^3^
0 restraints	Δρ_min_ = −0.18 e Å^−^^3^

### Special details

**Table:**

Geometry. All e.s.d.'s (except the e.s.d. in the dihedral angle between two l.s. planes) are estimated using the full covariance matrix. The cell e.s.d.'s are taken into account individually in the estimation of e.s.d.'s in distances, angles and torsion angles; correlations between e.s.d.'s in cell parameters are only used when they are defined by crystal symmetry. An approximate (isotropic) treatment of cell e.s.d.'s is used for estimating e.s.d.'s involving l.s. planes.
Refinement. Refinement of *F*^2^ against ALL reflections. The weighted *R*-factor *wR* and goodness of fit *S* are based on *F*^2^, conventional *R*-factors *R* are based on *F*, with *F* set to zero for negative *F*^2^. The threshold expression of *F*^2^ > σ(*F*^2^) is used only for calculating *R*-factors(gt) *etc*. and is not relevant to the choice of reflections for refinement. *R*-factors based on *F*^2^ are statistically about twice as large as those based on *F*, and *R*- factors based on ALL data will be even larger.

### Fractional atomic coordinates and isotropic or equivalent isotropic displacement parameters (Å^2^)

**Table:**

	*x*	*y*	*z*	*U*_iso_*/*U*_eq_	
Ni1	0.328740 (14)	0.17883 (3)	0.575762 (10)	0.01636 (7)	
C1	0.31560 (12)	0.2378 (2)	0.75097 (8)	0.0200 (3)	
H1A	0.3556	0.1440	0.7845	0.024*	
H1B	0.2635	0.3081	0.7850	0.024*	
C2	0.40253 (12)	0.3781 (2)	0.71795 (9)	0.0228 (3)	
C3	0.13224 (12)	0.2211 (2)	0.67502 (9)	0.0201 (3)	
H3A	0.0979	0.2483	0.7264	0.024*	
H3B	0.0831	0.1308	0.6462	0.024*	
C4	0.14153 (12)	0.4066 (2)	0.62701 (8)	0.0199 (3)	
H4A	0.0659	0.4615	0.6182	0.024*	
H4B	0.1878	0.4994	0.6568	0.024*	
C5	0.11558 (13)	0.2632 (2)	0.49152 (9)	0.0214 (3)	
H5A	0.1084	0.3382	0.4425	0.026*	
H5B	0.0404	0.2557	0.5150	0.026*	
C6	0.15460 (13)	0.0613 (2)	0.47048 (9)	0.0235 (3)	
C7	0.24547 (12)	0.5370 (2)	0.51157 (9)	0.0201 (3)	
H7A	0.2688	0.6278	0.5531	0.024*	
H7B	0.1889	0.5995	0.4769	0.024*	
C8	0.34815 (11)	0.4803 (2)	0.46292 (8)	0.0190 (3)	
C9	0.24045 (13)	−0.0747 (2)	0.70537 (9)	0.0226 (3)	
H9A	0.3177	−0.1259	0.7083	0.027*	
H9B	0.2015	−0.1366	0.6603	0.027*	
C10	0.17986 (12)	−0.1308 (2)	0.78149 (8)	0.0205 (3)	
N1	0.24747 (9)	0.13322 (17)	0.68850 (7)	0.0161 (2)	
N2	0.19470 (10)	0.36547 (16)	0.54870 (7)	0.0155 (2)	
O1	0.37976 (9)	0.61360 (17)	0.41363 (7)	0.0304 (3)	
H1	0.4430	0.5870	0.3962	0.046*	
O2	0.45601 (11)	0.4832 (2)	0.76438 (7)	0.0434 (3)	
O3	0.18039 (11)	−0.31910 (15)	0.79037 (7)	0.0333 (3)	
H3	0.1531	−0.3471	0.8339	0.050*	
O4	0.13713 (9)	−0.01849 (16)	0.82733 (6)	0.0293 (3)	
O5	0.09695 (7)	−0.02364 (13)	0.41745 (5)	0.0374 (3)	
O6	0.45974 (7)	−0.01221 (13)	0.58721 (5)	0.0402 (3)	
H6A	0.4811	−0.1050	0.5547	0.060*	
H6B	0.4970	−0.0318	0.6315	0.060*	
O7	0.41759 (8)	0.37646 (16)	0.64146 (6)	0.0234 (2)	
O8	0.23968 (9)	−0.00858 (15)	0.50781 (6)	0.0280 (2)	
O9	0.39642 (9)	0.32508 (15)	0.47244 (6)	0.0232 (2)	

### Atomic displacement parameters (Å^2^)

**Table:**

	*U*^11^	*U*^22^	*U*^33^	*U*^12^	*U*^13^	*U*^23^
Ni1	0.01745 (10)	0.01728 (10)	0.01441 (10)	0.00186 (7)	0.00174 (6)	−0.00042 (7)
C1	0.0201 (7)	0.0249 (8)	0.0149 (7)	−0.0024 (6)	−0.0003 (5)	−0.0006 (6)
C2	0.0189 (7)	0.0290 (8)	0.0205 (7)	−0.0038 (6)	−0.0002 (6)	−0.0009 (6)
C3	0.0146 (6)	0.0271 (8)	0.0187 (7)	−0.0010 (6)	0.0028 (5)	0.0029 (6)
C4	0.0194 (7)	0.0231 (8)	0.0172 (7)	0.0046 (6)	0.0031 (5)	−0.0012 (6)
C5	0.0212 (7)	0.0236 (8)	0.0190 (7)	−0.0046 (6)	−0.0042 (6)	0.0006 (6)
C6	0.0257 (8)	0.0259 (8)	0.0191 (7)	−0.0088 (6)	0.0063 (6)	−0.0056 (6)
C7	0.0218 (7)	0.0150 (7)	0.0236 (7)	−0.0007 (6)	0.0033 (6)	0.0014 (6)
C8	0.0179 (7)	0.0226 (8)	0.0165 (7)	−0.0058 (6)	−0.0020 (5)	0.0006 (6)
C9	0.0312 (8)	0.0165 (8)	0.0203 (7)	−0.0005 (6)	0.0067 (6)	0.0000 (6)
C10	0.0228 (7)	0.0214 (8)	0.0172 (7)	−0.0028 (6)	−0.0009 (6)	0.0000 (6)
N1	0.0177 (6)	0.0157 (6)	0.0151 (6)	−0.0010 (4)	0.0018 (4)	−0.0003 (5)
N2	0.0170 (5)	0.0144 (6)	0.0150 (5)	−0.0014 (4)	0.0010 (4)	−0.0014 (4)
O1	0.0223 (5)	0.0333 (6)	0.0359 (6)	−0.0026 (5)	0.0076 (5)	0.0145 (5)
O2	0.0451 (7)	0.0591 (9)	0.0260 (6)	−0.0309 (7)	−0.0008 (5)	−0.0090 (6)
O3	0.0559 (8)	0.0203 (6)	0.0240 (6)	−0.0024 (5)	0.0132 (5)	0.0044 (5)
O4	0.0390 (6)	0.0272 (6)	0.0222 (5)	0.0020 (5)	0.0111 (5)	−0.0003 (5)
O5	0.0315 (6)	0.0481 (8)	0.0327 (6)	−0.0110 (6)	0.0007 (5)	−0.0250 (6)
O6	0.0448 (7)	0.0493 (8)	0.0263 (6)	0.0301 (6)	−0.0022 (5)	−0.0052 (6)
O7	0.0203 (5)	0.0324 (6)	0.0176 (5)	−0.0079 (4)	0.0021 (4)	−0.0005 (4)
O8	0.0352 (6)	0.0183 (5)	0.0304 (6)	0.0005 (5)	−0.0026 (5)	−0.0066 (5)
O9	0.0245 (5)	0.0244 (6)	0.0210 (5)	0.0030 (4)	0.0060 (4)	0.0019 (4)

### Geometric parameters (Å, °)

**Table:**

Ni1—O8	2.0006 (10)	C5—H5A	0.9700
Ni1—O7	2.0260 (10)	C5—H5B	0.9700
Ni1—O6	2.0300 (9)	C6—O5	1.2468 (16)
Ni1—N2	2.0738 (11)	C6—O8	1.2582 (19)
Ni1—N1	2.1421 (11)	C7—N2	1.4708 (17)
Ni1—O9	2.1591 (10)	C7—C8	1.5121 (19)
C1—N1	1.4852 (18)	C7—H7A	0.9700
C1—C2	1.517 (2)	C7—H7B	0.9700
C1—H1A	0.9700	C8—O9	1.2239 (17)
C1—H1B	0.9700	C8—O1	1.2945 (17)
C2—O2	1.2245 (18)	C9—N1	1.4717 (18)
C2—O7	1.2880 (17)	C9—C10	1.514 (2)
C3—N1	1.4903 (18)	C9—H9A	0.9700
C3—C4	1.519 (2)	C9—H9B	0.9700
C3—H3A	0.9700	C10—O4	1.2054 (17)
C3—H3B	0.9700	C10—O3	1.3140 (18)
C4—N2	1.4818 (17)	O1—H1	0.8200
C4—H4A	0.9700	O3—H3	0.8200
C4—H4B	0.9700	O6—H6A	0.880
C5—N2	1.4922 (17)	O6—H6B	0.859
C5—C6	1.516 (2)		
			
O8—Ni1—O7	177.83 (4)	O5—C6—O8	125.26 (14)
O8—Ni1—O6	90.65 (4)	O5—C6—C5	116.05 (13)
O7—Ni1—O6	90.80 (4)	O8—C6—C5	118.68 (13)
O8—Ni1—N2	84.33 (4)	N2—C7—C8	110.17 (11)
O7—Ni1—N2	94.08 (4)	N2—C7—H7A	109.6
O6—Ni1—N2	172.75 (4)	C8—C7—H7A	109.6
O8—Ni1—N1	99.45 (4)	N2—C7—H7B	109.6
O7—Ni1—N1	81.88 (4)	C8—C7—H7B	109.6
O6—Ni1—N1	99.65 (4)	H7A—C7—H7B	108.1
N2—Ni1—N1	86.36 (4)	O9—C8—O1	125.02 (13)
O8—Ni1—O9	92.86 (4)	O9—C8—C7	121.86 (13)
O7—Ni1—O9	85.40 (4)	O1—C8—C7	113.09 (13)
O6—Ni1—O9	95.41 (4)	N1—C9—C10	116.13 (12)
N2—Ni1—O9	79.67 (4)	N1—C9—H9A	108.3
N1—Ni1—O9	160.37 (4)	C10—C9—H9A	108.3
N1—C1—C2	114.38 (11)	N1—C9—H9B	108.3
N1—C1—H1A	108.7	C10—C9—H9B	108.3
C2—C1—H1A	108.7	H9A—C9—H9B	107.4
N1—C1—H1B	108.7	O4—C10—O3	124.91 (14)
C2—C1—H1B	108.7	O4—C10—C9	124.69 (13)
H1A—C1—H1B	107.6	O3—C10—C9	110.40 (12)
O2—C2—O7	123.39 (14)	C9—N1—C1	112.09 (11)
O2—C2—C1	119.36 (13)	C9—N1—C3	112.08 (11)
O7—C2—C1	117.23 (13)	C1—N1—C3	112.03 (11)
N1—C3—C4	110.60 (11)	C9—N1—Ni1	109.82 (8)
N1—C3—H3A	109.5	C1—N1—Ni1	107.45 (8)
C4—C3—H3A	109.5	C3—N1—Ni1	102.85 (8)
N1—C3—H3B	109.5	C7—N2—C4	113.14 (11)
C4—C3—H3B	109.5	C7—N2—C5	111.51 (11)
H3A—C3—H3B	108.1	C4—N2—C5	112.76 (11)
N2—C4—C3	109.56 (12)	C7—N2—Ni1	106.69 (8)
N2—C4—H4A	109.8	C4—N2—Ni1	104.89 (8)
C3—C4—H4A	109.8	C5—N2—Ni1	107.26 (8)
N2—C4—H4B	109.8	C8—O1—H1	109.5
C3—C4—H4B	109.8	C10—O3—H3	109.5
H4A—C4—H4B	108.2	Ni1—O6—H6A	129.9
N2—C5—C6	113.66 (12)	Ni1—O6—H6B	123.5
N2—C5—H5A	108.8	H6A—O6—H6B	105.4
C6—C5—H5A	108.8	C2—O7—Ni1	117.40 (9)
N2—C5—H5B	108.8	C6—O8—Ni1	115.17 (10)
C6—C5—H5B	108.8	C8—O9—Ni1	109.95 (9)
H5A—C5—H5B	107.7		
			
N1—C1—C2—O2	173.79 (14)	C3—C4—N2—C5	73.50 (14)
N1—C1—C2—O7	−7.7 (2)	C3—C4—N2—Ni1	−42.89 (12)
N1—C3—C4—N2	58.79 (15)	C6—C5—N2—C7	116.31 (13)
N2—C5—C6—O5	−173.79 (12)	C6—C5—N2—C4	−115.13 (13)
N2—C5—C6—O8	7.39 (19)	C6—C5—N2—Ni1	−0.16 (13)
N2—C7—C8—O9	−17.75 (18)	O8—Ni1—N2—C7	−123.50 (9)
N2—C7—C8—O1	164.25 (12)	O7—Ni1—N2—C7	55.03 (9)
N1—C9—C10—O4	0.8 (2)	N1—Ni1—N2—C7	136.61 (9)
N1—C9—C10—O3	−179.23 (13)	O9—Ni1—N2—C7	−29.54 (8)
C10—C9—N1—C1	63.40 (16)	O8—Ni1—N2—C4	116.22 (9)
C10—C9—N1—C3	−63.58 (16)	O7—Ni1—N2—C4	−65.25 (9)
C10—C9—N1—Ni1	−177.24 (10)	N1—Ni1—N2—C4	16.33 (9)
C2—C1—N1—C9	133.66 (13)	O9—Ni1—N2—C4	−149.82 (9)
C2—C1—N1—C3	−99.34 (14)	O8—Ni1—N2—C5	−3.90 (8)
C2—C1—N1—Ni1	12.92 (14)	O7—Ni1—N2—C5	174.63 (8)
C4—C3—N1—C9	−157.70 (11)	N1—Ni1—N2—C5	−103.78 (9)
C4—C3—N1—C1	75.29 (14)	O9—Ni1—N2—C5	90.06 (9)
C4—C3—N1—Ni1	−39.81 (12)	O2—C2—O7—Ni1	175.79 (13)
O8—Ni1—N1—C9	48.50 (10)	C1—C2—O7—Ni1	−2.69 (18)
O7—Ni1—N1—C9	−133.23 (10)	O6—Ni1—O7—C2	−91.55 (10)
O6—Ni1—N1—C9	−43.79 (9)	N2—Ni1—O7—C2	93.81 (11)
N2—Ni1—N1—C9	132.13 (10)	N1—Ni1—O7—C2	8.08 (11)
O9—Ni1—N1—C9	176.63 (11)	O9—Ni1—O7—C2	173.09 (11)
O8—Ni1—N1—C1	170.66 (9)	O5—C6—O8—Ni1	170.31 (11)
O7—Ni1—N1—C1	−11.06 (9)	C5—C6—O8—Ni1	−10.99 (17)
O6—Ni1—N1—C1	78.38 (8)	O6—Ni1—O8—C6	−166.26 (10)
N2—Ni1—N1—C1	−105.71 (9)	N2—Ni1—O8—C6	8.50 (10)
O9—Ni1—N1—C1	−61.20 (17)	N1—Ni1—O8—C6	93.84 (11)
O8—Ni1—N1—C3	−70.97 (9)	O9—Ni1—O8—C6	−70.82 (11)
O7—Ni1—N1—C3	107.30 (9)	O1—C8—O9—Ni1	169.74 (12)
O6—Ni1—N1—C3	−163.26 (8)	C7—C8—O9—Ni1	−8.00 (16)
N2—Ni1—N1—C3	12.65 (8)	O8—Ni1—O9—C8	105.34 (9)
O9—Ni1—N1—C3	57.16 (16)	O7—Ni1—O9—C8	−73.36 (9)
C8—C7—N2—C4	148.46 (11)	O6—Ni1—O9—C8	−163.74 (9)
C8—C7—N2—C5	−83.18 (14)	N2—Ni1—O9—C8	21.64 (9)
C8—C7—N2—Ni1	33.64 (12)	N1—Ni1—O9—C8	−23.69 (18)
C3—C4—N2—C7	−158.79 (11)		

### Hydrogen-bond geometry (Å, °)

**Table:**

*D*—H···*A*	*D*—H	H···*A*	*D*···*A*	*D*—H···*A*
O1—H1···O7^i^	0.82	1.77	2.5557 (14)	159
O3—H3···O5^ii^	0.82	1.79	2.5864 (13)	164
O6—H6A···O9^iii^	0.88	2.15	2.9294 (12)	148
O6—H6B···O2^iv^	0.86	1.81	2.6394 (11)	162

Symmetry codes: (i) −*x*+1, −*y*+1, −*z*+1; (ii) *x*, −*y*−1/2, *z*+1/2; (iii) −*x*+1, −*y*, −*z*+1; (iv) −*x*+1, *y*−1/2, −*z*+3/2.
